# Targeting Heme Oxygenase-1 in Cardiovascular and Kidney Disease

**DOI:** 10.3390/antiox8060181

**Published:** 2019-06-18

**Authors:** Heather A. Drummond, Zachary L. Mitchell, Nader G. Abraham, David E. Stec

**Affiliations:** 1 Department of Physiology and Biophysics, Mississippi Center for Obesity Research, University of Mississippi Medical Center, Jackson, MI 39216, USA; hdrummond@umc.edu (H.A.D.); zmitchell@umc.edu (Z.L.M.); 2 Departments of Medicine and Pharmacology, New York Medical College, Vahalla, NY 10595, USA; Nader_Abraham@nymc.edu; 3 Joan C. Edwards School of Medicine, Marshall University, Huntington, VA 25701, USA

**Keywords:** bilirubin, carbon monoxide, hypertension, kidney injury, blood pressure

## Abstract

Heme oxygenase (HO) plays an important role in the cardiovascular system. It is involved in many physiological and pathophysiological processes in all organs of the cardiovascular system. From the regulation of blood pressure and blood flow to the adaptive response to end-organ injury, HO plays a critical role in the ability of the cardiovascular system to respond and adapt to changes in homeostasis. There have been great advances in our understanding of the role of HO in the regulation of blood pressure and target organ injury in the last decade. Results from these studies demonstrate that targeting of the HO system could provide novel therapeutic opportunities for the treatment of several cardiovascular and renal diseases. The goal of this review is to highlight the important role of HO in the regulation of cardiovascular and renal function and protection from disease and to highlight areas in which targeting of the HO system needs to be translated to help benefit patient populations.

## 1. Introduction

Heme oxygenase (HO) is the rate-limiting enzyme in the breakdown of heme in the body. Two major isoforms of HO exist: heme oxygenase-1 (HO-1) and heme oxygenase-2 (HO-2). Both isoforms of HO are expressed in all tissues of the body. HO-1 is the inducible isoform whose expression is regulated by a wide variety of physiologic and pathophysiologic stimuli [1]. Heme oxygenase-2 is the constitutive isoform of HO found in all tissues and cells of the body [2]. Bilirubin, an endogenous antioxidant derived from HO catabolism of heme is capable of directly scavenging reactive oxygen species (ROS) and inhibiting oxidative stress [3,4,5,6,7]. Carbon monoxide (CO), the other product derived from HO catabolism of heme, is a gaseous transmitter which can affect ion channels, nitric oxide release, as well as mitochondrial proteins [8,9]. An underappreciated aspect of HO breakdown of heme is the release of free iron and its subsequent sequestration by induction of ferritin. Labile iron is toxic due to its ability to release ROS which can then result in cellular damage especially in renal tubule cells [10]. Ferritin is a cytoprotective protein that is also involved in the regulation of myelopoiesis and inflammation [11]. HO-1 induction mediates many beneficial effects in the cardiovascular system and kidney as well as in metabolism [12,13,14]. For this review, we will focus on the role of HO in the cardiovascular system and the potential for targeting this system for the treatment of cardiovascular and end-organ damage. 

## 2. HO and the Cardiovascular System

### 2.1. Role of Heme Oxygenase in the Regulation of Blood Pressure

The anti-hypertensive actions of HO-1 induction was first demonstrated in the spontaneously hypertensive rat (SHR) treated with the HO-1 inducers, stannous chloride (SnCl2) and hemin [15,16,17,18]. Later studies using genetic overexpression of HO-1 demonstrated similar anti-hypertensive effects in this model [19,20]. HO-1 induction has also been demonstrated to have anti-hypertensive effects in other models of hypertension such as angiotensin-II (Ang II)-dependent hypertension, deoxycorticosterone acetate (DOCA)-salt hypertension, and renovascular hypertension [21,22,23,24]. Likewise, deficiency in HO-1 exacerbates the blood pressure response to Ang II-dependent hypertension and DOCA-salt hypertension [25,26].

**Figure 1:**
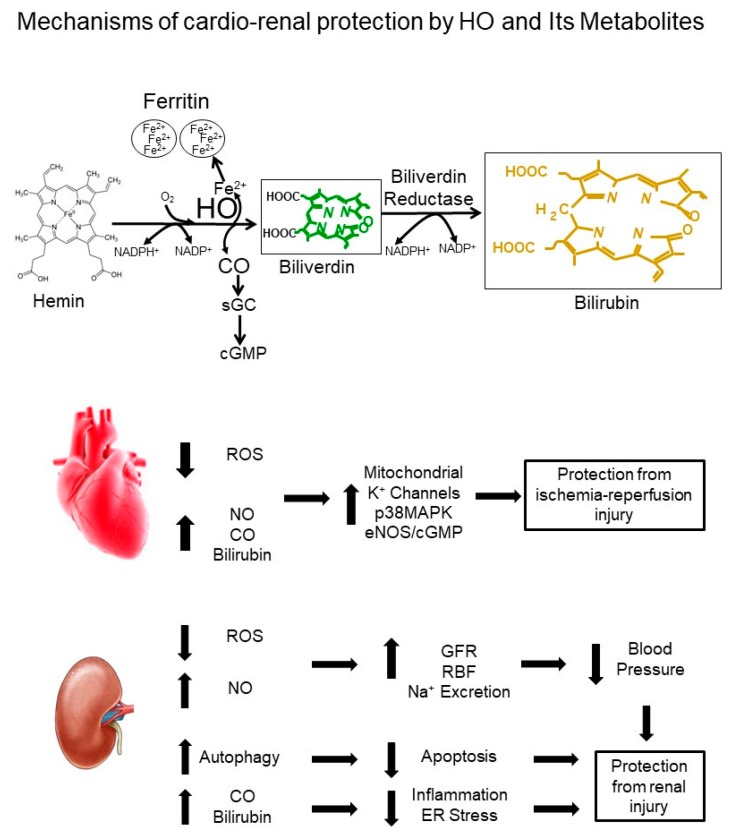
Proposed mechanisms by which HO and its metabolites confer cardio-renal protection.

While global induction of HO-1 had been repeatedly demonstrated to lower blood pressure in several different models of hypertension, the role of the kidney in this response was not known. However, studies in which HO-1 was induced specifically in the kidney with the known inducer cobalt-protoporphyrin (CoPP) or genetically, via kidney-specific overexpression of HO-1, have demonstrated the anti-hypertensive effects of HO-1 in the kidney [27,28]. One mechanism by which kidney-specific induction of HO-1 lowers blood pressure in Ang II-dependent hypertension is by decreasing reactive oxygen species (ROS) generation (Figure 1) [27,29,30]. Further studies in cultured renal tubule cells have also demonstrated the antioxidant actions of HO-1 overexpression against enhanced Ang II-mediated ROS production [31,32]. Additional studies found that increases in renal perfusion pressure upregulate HO-1 levels and that renal medullary inhibition of HO-1 results in the development of salt-sensitive hypertension [33].

The role of heme oxygenase-2 (HO-2) in the regulation of blood pressure has not been studied to the extent of HO-1. Mice deficient for HO-2 do not exhibit enhanced blood pressure response to Ang II-dependent or N(ω)-nitro-L-arginine methyl ester (*L*-*NAME*)-dependent hypertension; however, HO-2 knockout mice do exhibit a sex difference in response to renovascular hypertension with male knockout mice exhibiting an exaggerated blood pressure response as compared to wild-type mice and female knockout mice lacking any difference to wild-type mice [34,35]. 

Heme oxygenase can also be induced by natural products such as curcumin, flavonoids, isothiocyanates and organosulfur compounds such as diallyl sulfide (DAS) and its other derivatives [36]. Curcumin lowers blood pressure in many models of experimental hypertension including Ang II-dependent hypertension through alterations in angiotensin receptor 1 (AT1R) levels [37]. Curcumin also improves blood pressure through its effects on vascular function as well as its anti-inflammatory actions [38,39]. Regular consumption of flavonoids exerts beneficial cardiovascular effects and may reduce the onset or progression of hypertension; however, the mechanism by which this occurs is not fully understood although the anti-inflammatory actions of flavonoids may contribute to this beneficial effect [40,41]. While it is clear that natural HO inducing compounds can have protective antihypertensive and cardiovascular effects, the specific role of HO-1 induction in these effects has yet to be determined. 

Overall, induction of HO-1 results in lowering of blood pressure in several different models of experimental and genetic hypertension. One mechanism by which induction of HO-1 lowers blood pressure is through decreasing ROS production which is common in many forms of experimental hypertension [42,43]. The therapeutic potential of HO induction has been clearly shown in several models of experiments hypertension. One of the most impressive studies demonstrated that induction of HO-1 with a hemin pump resulted in a sustained reduction in blood pressure long after initial hemin treatment [44]. This study suggests that short-term induction of HO-1 may chronically lower blood pressure which could be a novel therapeutic approach to circumvent patient compliance issues faced when taking daily anti-hypertensive medications. One limitation of the preclinical studies to date is lack of experimental evidence for the anti-hypertensive actions of HO-1 induction in large animal models of hypertension. This limitation needs to be addressed so that these encouraging results can be directly translated to hypertensive patients. 

### 2.2. Role of Heme Oxygenase in the Regulation of Renal Function

Renal vascular and tubular function are both regulated by the expression of HO-1 and HO-2 since both of these isoforms are found throughout the kidney [45,46]. In the renal vasculature, HO-2 protects against excessive renal vasoconstriction through the generation of CO [47,48,49,50,51]. However, CO can also elicit renal vasoconstriction through increased reactive oxygen species (ROS) production in renal arterioles [52]. CO can also affect renal vascular tone through its complex interaction with nitric oxide (NO). Inhibition of NO production increases renal vascular CO production and inhibition of NO enhances renal vasoconstriction following HO inhibition [53,54]. However, vascular smooth muscle-specific overexpression of HO-1 results in an attenuation of NO-mediated vasorelaxation and hypertension suggesting that large increases in HO-mediated CO production can alter NO responsiveness [55]. These studies highlight the complex relationship between vascular CO and NO production. The other HO metabolite, bilirubin, has also been shown to have effects on renal vascular function. Mice made moderately hyperbilirubinemic via antagonism of hepatic UDP-glucuronosyltransferase 1-1 (UGT1A1) are resistant to Ang II-induced hypertension and decreases in renal blood flow and glomerular filtration rate (GFR) [56,57]. One mechanism by which bilirubin protects against Ang II-induced vasoconstriction is through the preservation of NO bioavailability [58,59]. Bilirubin preserves NO bioavailability due to its antioxidant actions which likely prevents the reaction of NO with superoxide anion and reduces subsequent peroxynitrite formation [60,61,62]. 

Arterial pressure regulates renal tubular function through the renal pressure-natriuretic response [63,64]. Renal perfusion pressure can directly alter HO activity in the renal medulla, and blockade of renal medullary HO activity significantly attenuates renal pressure natriuresis and results in salt-sensitive hypertension [33]. Renal tubular induction of HO-1 was found to increase sodium and water excretion as well as increase GFR [65,66]. However, the mechanism by which this occurs and the metabolites responsible for mediating these changes are not fully understood. Previous studies in both cultured thick ascending loop of Henle (TALH) cells and in transgenic mice specifically overexpressing HO-1 in the TALH have demonstrated that HO-1 can protect against Ang II-mediated increases in ROS production, decrease the levels of the sodium-potassium 2 chloride (NKCC2) transporter, and attenuate the development of Ang II-dependent hypertension [28,31]. HO induction stimulates the apical 70-pS K+ channel in the TALH through the generation of CO but not biliverdin/bilirubin [67]. Additional studies have suggested that HO derived CO is involved in the regulation of the epithelial sodium channel under conditions of hypoxia, but the physiological significance of this regulatory pathway is not clear [68]. 

## 3. HO and Target Organ Injury

### 3.1. HO and the Heart

HO has been shown to play a protective role in the heart in many different pathological conditions. HO-1 induction or overexpression has been demonstrated to protect against myocyte hypertrophy both in vitro and in vivo [24,69]. However, the role of HO induction in Ang II-dependent cardiac hypertrophy has been controversial with studies demonstrating both protection and lack of protection against Ang II-dependent cardiac hypertrophy [70,71]. While the role of HO induction in preventing Ang II-dependent hypertrophy is controversial, both bilirubin and CO administered alone protect against Ang II-dependent cardiac hypertrophy [70,72]. 

Alterations in the HO system have shown the most promise in the protection of the heart against ischemia–reperfusion (IR) injury. Cardiac IR injury results in the upregulation of HO-1 and lack of this upregulation has been shown to contribute to ventricular fibrillation (VF) in diabetic animals [73,74]. Gene-targeted mice lacking HO-1 are more susceptible to cardiac IR injury, and transgenic overexpression of HO-1 in the heart protects against IR injury [75,76]. One mechanism by which HO protects against cardiac IR injury is through reductions in post-ischemia ROS production (Figure 1) [75]. The decrease in post-ischemic ROS production by HO induction likely occurs through increased bilirubin production. Several studies have demonstrated bilirubin itself protects the heart from IR injury [77,78,79]. A potential therapeutic strategy utilizing HO-1 induction to protect against IR injury is the design of “smart” gene therapy vectors in which expression of HO-1 is regulated in a hypoxia-specific fashion [80,81]. In this system, HO-1 expression is regulated by hypoxia-specific transcription factors which allow high levels of HO-1 expression during the ischemic period but “turn-off” HO-1 expression once the hypoxic signal has ended [80,81]. CO, administered via carbon-monoxide releasing molecules (CORMs) or inhaled, has been shown to protect the heart against IR injury [82,83,84,85]. CO acts through several different mechanisms including mitochondrial potassium channels, activation of the p38 mitogen-activated protein kinase (p38MAPK) pathway, and activation of endothelial nitric oxide synthase (eNOS)/cGMP pathway (Figure 1) [82,83]. 

While there is an abundance of experimental evidence from rodent models, the translation of the protective effect of HO induction has not been significantly explored in large animal preclinical models as well as in human patients. In swine, a model of HO-1 overexpression exists; however, the protective actions of HO-1 in the heart of this model have not been studied [86]. Human HO-1 overexpression driven by a recombinant adenoassociated virus (rAAV) is effective in attenuating post-ischemic inflammation and preserving cardiac function in a swine model [87]. In humans, a (GT)n dinucleotide length polymorphism in the promoter of the HO-1 gene regulates its inducibility. Short (class S) repeats are associated with greater up-regulation of HO-1 than are long repeats. Class S repeats are protective against the incidence of ischemic coronary heart disease and cardiac allograft vasculopathy in several populations [88,89,90]. It is clear that more studies are needed in large animal preclinical models as well as in human patient populations to fully translate the wealth of data that has emerged on the protective actions of HO-1 induction in small animal models. 

### 3.2. HO and the Kidney

HO-1 is expressed at low levels in the kidney under basal conditions. However, it can be induced in several clinically relevant renal disease states, including hypertension, several forms of acute renal injury, and transplant rejection. The protective role for HO-1 in models of acute kidney injury has been elucidated using chemical inducers and inhibitors of HO-1 with results of these studies further corroborated in HO-1 knockout or renal cell-type specific HO-1 overexpression. Human HO-1 deficiency mirrors the renal injury reported in the HO-1 knockout mouse including renal iron deposition, increased renal oxidative stress and the development of nephritis, further demonstrating the important protective role of this system in the kidney [91,92]. 

Renal IR injury occurs after blood flow to the kidney is reduced for an extended period. It can lead to progressive loss of renal function following the reestablishment of blood flow due to injury to specific cell types which require a constant source of oxygen for their long-term survival. Induction of HO-1 with hemin or tin-chloride protects against renal IR injury [93,94]. HO-1 induction also protects against other forms of acute kidney injury brought on by nephrotoxins such as glycerol and cisplatin [95,96,97,98]. Additional studies have demonstrated that deletion HO-1 specifically in the proximal tubule of the kidney worsens cisplatin-induced acute kidney injury through regulation of cleaved caspase-3 and modulation of p38 signaling while overexpression of HO-1 in the proximal tubule protects against cisplatin-induced acute renal injury by decreasing the levels of cleaved caspase-3 [99]. Another mechanism by which HO-1 protects the kidney is through regulation of autophagy (Figure 1). Autophagy is a complex process which can constitute a stress adaptation that avoids cell death (and suppresses apoptosis), whereas, in other cellular settings, it constitutes an alternative cell-death pathway. Renal tubule cells lacking HO-1 exhibit higher levels of basal autophagy, impaired progression of autophagy, and increased apoptosis after cisplatin treatment which is reversed upon restoration of HO-1 expression [100]. 

In humans, patients with the long (GT)n dinucleotide length polymorphism in the promoter of the HO-1 gene (decreased expression), have 1.58 fold higher odds of acute kidney injury (AKI) following cardiac surgery as compared to individuals with the short repeat polymorphism [101]. In human AKI patients, plasma and urinary HO-1 levels correlate with renal HO-1 expression and degree of renal injury indicating that levels of HO-1 could be a viable biomarker for AKI [102]. However, the source, as well as the function of plasma HO-1 in the setting of AKI, is not known. For example, is HO-1 found in the plasma enzymatically active? If so, what is its substrate? If not, what is its function? These questions need to be addressed in order to determine the role of circulating HO-1 in AKI. 

Not only has the protective actions of HO-1 induction been studied in the setting of AKI, the specific role of its major metabolites, bilirubin, and CO have also been investigated. Bilirubin treatment has had mixed effects in rat models of renal IR-injury with several studies demonstrating complete protection and one study demonstrating partial protection [93,103,104]. However, several studies in both human patient populations and in the hyperbilirubinemic Gunn rat demonstrate protection against the development of diabetic kidney injury [60,105]. Human studies have focused on the correlation of serum bilirubin levels with the development of acute renal injury. Bilirubin treatment has not yet been tried as a therapy against acute kidney injury in humans. Bilirubin has traditionally been thought to protect the kidney through its anti-oxidant and anti-inflammatory properties; however, new studies have identified bilirubin as an activator of nuclear hormone receptors [7,106]. This signaling property of bilirubin raises the possibility that, in addition to its potent anti-oxidant actions, bilirubin may afford renoprotection through alterations in metabolism which may help renal tubule cells recover after injury. 

Carbon monoxide has also been demonstrated to protect the kidney against acute injury as well as increase renal allograft survival and viability following transplantation [107,108]. CO donor drugs have been demonstrated to reduce renal injury following ischemia as well as nephrotoxin exposure [109,110,111]. CO inhalation therapy has also been effective in protecting the kidney against IR injury as well as playing a beneficial role in renal allograft survival [108,112,113,114]. CO exerts its protective effects through alterations in several signaling mechanisms including: (1) vascular endothelial growth factor (VEGF) production; (2) the inflammatory response through regulation of high-mobility group box 1 (HMGB1) proteins; (3) through the endoplasmatic reticulum (ER) stress response; (4) through metabolic hormones such as fibroblast growth factor-21 (FGF21); and (5) increased expression of the circadian rhythm protein Period 2 (Per2) [115,116,117,118,119]. CO inhalation has also proven to be a successful therapy in protecting the kidney against IR injury in swine models, which are highly translational to humans [107,114,120]. 

## 4. Conclusions

The heme oxygenase system provides many opportunities for protection against cardiovascular and renal disease (Figure 1). Several strategies to induce HO-1 apart from the traditional porphyrin-based compounds and heme have been reported. These include targeting of the nuclear factor (erythroid-derived 2)-like 2 (NFE2L2 and Nrf2, respectively) [121,122,123], and treatment with resveratrol and electroacupuncture [124,125]. One area in which research into the protective actions of HO induction is lacking is in large animal models of cardiovascular and renal disease. This is especially true for studies examining the anti-hypertensive actions of HO-1 induction. One potential reason for this has been the lack of adequate experimental drugs to allow for induction or inhibition of HO in larger animals. The development of novel HO inducers described above, as well as novel non-porphyrin based HO inhibitors, will help in this area [126,127,128]. With the rapid development of CRISPR/Cas9 technology, it is now possible to alter HO-1 levels in larger preclinical animal models which will further advance the therapeutic potential of the HO system for the treatment of cardiovascular and renal disease. 

## References

[1] Abraham N.G., Kappas A. (2008). Pharmacological and clinical aspects of heme oxygenase. Pharmacol. Rev..

[2] McCoubrey W.K., Maines M.D. (1994). The structure, organization and differential expression of the gene encoding rat heme oxygenase-2. Gene.

[3] Stocker R., Yamamoto Y., McDonagh A.F., Glazer A.N., Ames B.N. (1987). Bilirubin is an antioxidant of possible physiological importance. Science.

[4] Stocker R., Peterhans E. (1989). Antioxidant properties of conjugated bilirubin and biliverdin: Biologically relevant scavenging of hypochlorous acid. Free Radic. Res. Commun..

[5] Lanone S., Bloc S., Foresti R., Almolki A., Taille C., Callebert J., Conti M., Goven D., Aubier M., Dureuil B. (2005). Bilirubin decreases nos2 expression via inhibition of NAD(P)H oxidase: Implications for protection against endotoxic shock in rats. FASEB J..

[6] Sundararaghavan V.L., Binepal S., Stec D.E., Sindhwani P., Hinds T.D. (2018). Bilirubin, a new therapeutic for kidney transplant?. Transplant. Rev. (Orlando).

[7] Hinds T.D., Stec D.E. (2018). Bilirubin, a Cardiometabolic Signaling Molecule. Hypertension.

[8] Cebova M., Kosutova M., Pechanova O. (2016). Cardiovascular effects of gasotransmitter donors. Physiol. Res..

[9] Motterlini R., Foresti R. (2017). Biological signaling by carbon monoxide and carbon monoxide-releasing molecules. Am. J. Physiol. Cell Physiol..

[10] Scindia Y., Leeds J., Swaminathan S. (2019). Iron Homeostasis in Healthy Kidney and its Role in Acute Kidney Injury. Semin. Nephrol..

[11] Koorts A.M., Viljoen M. (2007). Ferritin and ferritin isoforms I: Structure-function relationships, synthesis, degradation and secretion. Arch. Physiol. Biochem..

[12] Abraham N.G., Kappas A. (2005). Heme oxygenase and the cardiovascular-renal system. Free Radic. Biol. Med..

[13] Hosick P.A., Stec D.E. (2012). Heme oxygenase, a novel target for the treatment of hypertension and obesity?. Am. J. Physiol. Regul. Integr. Comp. Physiol..

[14] Raffaele M., Li Volti G., Barbagallo I.A., Vanella L. (2016). Therapeutic Efficacy of Stem Cells Transplantation in Diabetes: Role of Heme Oxygenase. Front. Cell Dev. Biol..

[15] Sacerdoti D., Escalante B., Abraham N.G., McGiff J.C., Levere R.D., Schwartzman M.L. (1989). Treatment with tin prevents the development of hypertension in spontaneously hypertensive rats. Science.

[16] Levere R.D., Martasek P., Escalante B., Schwartzman M.L., Abraham N.G. (1990). Effect of heme arginate administration on blood pressure in spontaneously hypertensive rats. J. Clin. Investig..

[17] Martasek P., Schwartzman M.L., Goodman A.I., Solangi K.B., Levere R.D., Abraham N.G. (1991). Hemin and L-arginine regulation of blood pressure in spontaneous hypertensive rats. J. Am. Soc. Nephrol..

[18] da Silva J.L., Tiefenthaler M., Park E., Escalante B., Schwartzman M.L., Levere R.D., Abraham N.G. (1994). Tin-mediated heme oxygenase gene activation and cytochrome P450 arachidonate hydroxylase inhibition in spontaneously hypertensive rats. Am. J. Med. Sci..

[19] Sabaawy H.E., Zhang F., Nguyen X., ElHosseiny A., Nasjletti A., Schwartzman M., Dennery P., Kappas A., Abraham N.G. (2001). Human heme oxygenase-1 gene transfer lowers blood pressure and promotes growth in spontaneously hypertensive rats. Hypertension.

[20] Goodman A.I., Quan S., Yang L., Synghal A., Abraham N.G. (2003). Functional expression of human heme oxygenase-1 gene in renal structure of spontaneously hypertensive rats. Exp. Biol. Med. (Maywood).

[21] Botros F.T., Schwartzman M.L., Stier C.T., Goodman A.I., Abraham N.G. (2005). Increase in heme oxygenase-1 levels ameliorates renovascular hypertension. Kidney Int..

[22] Vera T., Yanes L., Reckelhoff J.F., Stec D.E. (2005). Heme Oxygenase-1 Induction Prevents the Increase in Oxidative Stress in the Kidney of Angiotensin II Hypertensive Mice. Hypertension.

[23] Jadhav A., Torlakovic E., Ndisang J.F. (2009). Hemin therapy attenuates kidney injury in deoxycorticosterone acetate-salt hypertensive rats. Am. J. Physiol. Ren. Physiol..

[24] Jadhav A., Ndisang J.F. (2009). Heme arginate suppresses cardiac lesions and hypertrophy in deoxycorticosterone acetate-salt hypertension. Exp. Biol. Med. (Maywood).

[25] Nath K.A., d’Uscio L.V., Juncos J.P., Croatt A.J., Manriquez M.C., Pittock S.T., Katusic Z.S. (2007). An analysis of the DOCA-salt model of hypertension in HO-1-/- mice and the Gunn rat. Am. J. Physiol. Heart Circ. Physiol..

[26] Yang L., Quan S., Nasjletti A., Laniado-Schwartzman M., Abraham N.G. (2004). Heme oxygenase-1 gene expression modulates angiotensin II-induced increase in blood pressure. Hypertension.

[27] Vera T., Kelsen S., Stec D.E. (2008). Kidney-specific induction of heme oxygenase-1 prevents angiotensin II hypertension. Hypertension.

[28] Stec D.E., Drummond H.A., Gousette M.U., Storm M.V., Abraham N.G., Csongradi E. (2012). Expression of heme oxygenase-1 in thick ascending loop of henle attenuates angiotensin II-dependent hypertension. J. Am. Soc. Nephrol..

[29] Vera T., Kelsen S., Yanes L.L., Reckelhoff J.F., Stec D.E. (2007). HO-1 induction lowers blood pressure and superoxide production in the renal medulla of angiotensin II hypertensive mice. Am. J. Physiol. Regul. Integr. Comp. Physiol..

[30] Stec D.E., Juncos L.A., Granger J.P. (2016). Renal intramedullary infusion of tempol normalizes the blood pressure response to intrarenal blockade of heme oxygenase-1 in angiotensin II-dependent hypertension. J. Am. Soc. Hypertens..

[31] Quan S., Yang L., Shnouda S., Schwartzman M.L., Nasjletti A., Goodman A.I., Abraham N.G. (2004). Expression of human heme oxygenase-1 in the thick ascending limb attenuates angiotensin II-mediated increase in oxidative injury. Kidney Int..

[32] Kelsen S., Patel B.J., Parker L.B., Vera T., Rimoldi J.M., Gadepalli R.S., Drummond H.A., Stec D.E. (2008). Heme oxygenase attenuates angiotensin II-mediated superoxide production in cultured mouse thick ascending loop of Henle cells. Am. J. Physiol. Ren. Physiol..

[33] Li N., Yi F., Dos Santos E.A., Donley D.K., Li P.L. (2007). Role of renal medullary heme oxygenase in the regulation of pressure natriuresis and arterial blood pressure. Hypertension.

[34] Stec D.E., Vera T., Storm M.V., McLemore G.R., Ryan M.J. (2009). Blood pressure and renal blow flow responses in heme oxygenase-2 knockout mice. Am. J. Physiol. Regul. Integr. Comp. Physiol..

[35] Stout J.M., Gousset M.U., Drummond H.A., Gray W., Pruett B.E., Stec D.E. (2013). Sex-specific effects of heme oxygenase-2 deficiency on renovascular hypertension. J. Am. Soc. Hypertens..

[36] Li Volti G., Sacerdoti D., Di Giacomo C., Barcellona M.L., Scacco A., Murabito P., Biondi A., Basile F., Gazzolo D., Abella R. (2008). Natural heme oxygenase-1 inducers in hepatobiliary function. World J. Gastroenterol..

[37] Yao Y., Wang W., Li M., Ren H., Chen C., Wang J., Wang W.E., Yang J., Zeng C. (2016). Curcumin Exerts its Anti-hypertensive Effect by Down-regulating the AT1 Receptor in Vascular Smooth Muscle Cells. Sci. Rep..

[38] Lan C., Chen X., Zhang Y., Wang W., Wang W.E., Liu Y., Cai Y., Ren H., Zheng S., Zhou L. (2018). Curcumin prevents strokes in stroke-prone spontaneously hypertensive rats by improving vascular endothelial function. BMC Cardiovasc. Disord..

[39] Campbell N.K., Fitzgerald H.K., Malara A., Hambly R., Sweeney C.M., Kirby B., Fletcher J.M., Dunne A. (2018). Naturally derived Heme-Oxygenase 1 inducers attenuate inflammatory responses in human dendritic cells and T cells: Relevance for psoriasis treatment. Sci. Rep..

[40] Maaliki D., Shaito A.A., Pintus G., El-Yazbi A., Eid A.H. (2019). Flavonoids in hypertension: A brief review of the underlying mechanisms. Curr. Opin. Pharmacol..

[41] Cho B.O., Yin H.H., Park S.H., Byun E.B., Ha H.Y., Jang S.I. (2016). Anti-inflammatory activity of myricetin from Diospyros lotus through suppression of NF-kappaB and STAT1 activation and Nrf2-mediated HO-1 induction in lipopolysaccharide-stimulated RAW264.7 macrophages. Biosci. Biotechnol. Biochem..

[42] Jin L., Beswick R.A., Yamamoto T., Palmer T., Taylor T.A., Pollock J.S., Pollock D.M., Brands M.W., Webb R.C. (2006). Increased reactive oxygen species contributes to kidney injury in mineralocorticoid hypertensive rats. J. Physiol. Pharmacol..

[43] Taylor N.E., Glocka P., Liang M., Cowley A.W. (2006). NADPH oxidase in the renal medulla causes oxidative stress and contributes to salt-sensitive hypertension in Dahl S rats. Hypertension.

[44] Wang R., Shamloul R., Wang X., Meng Q., Wu L. (2006). Sustained normalization of high blood pressure in spontaneously hypertensive rats by implanted hemin pump. Hypertension.

[45] Zou A.P., Billington H., Su N., Cowley A.W. (2000). Expression and actions of heme oxygenase in the renal medulla of rats. Hypertension.

[46] Hu Y., Ma N., Yang M., Semba R. (1998). Expression and distribution of heme oxygenase-2 mRNA and protein in rat kidney. J. Histochem. Cytochem..

[47] Johnson R.A., Lavesa M., Askari B., Abraham N.G., Nasjletti A. (1995). A heme oxygenase product, presumably carbon monoxide, mediates a vasodepressor function in rats. Hypertension.

[48] Kozma F., Johnson R.A., Nasjletti A. (1997). Role of carbon monoxide in heme-induced vasodilation. Eur. J. Pharmacol..

[49] Kaide J.I., Zhang F., Wei Y., Jiang H., Yu C., Wang W.H., Balazy M., Abraham N.G., Nasjletti A. (2001). Carbon monoxide of vascular origin attenuates the sensitivity of renal arterial vessels to vasoconstrictors. J. Clin. Investig..

[50] Zhang F., Kaide J., Wei Y., Jiang H., Yu C., Balazy M., Abraham N.G., Wang W., Nasjletti A. (2001). Carbon monoxide produced by isolated arterioles attenuates pressure-induced vasoconstriction. Am. J. Physiol. Heart Circ. Physiol..

[51] Wang H., Garvin J.L., D’Ambrosio M.A., Falck J.R., Leung P., Liu R., Ren Y., Carretero O.A. (2011). Heme oxygenase metabolites inhibit tubuloglomerular feedback in vivo. Am. J. Physiol. Heart Circ. Physiol..

[52] Lamon B.D., Zhang F.F., Puri N., Brodsky S.V., Goligorsky M.S., Nasjletti A. (2009). Dual pathways of carbon monoxide-mediated vasoregulation: Modulation by redox mechanisms. Circ. Res..

[53] Rodriguez F., Lamon B.D., Gong W., Kemp R., Nasjletti A. (2004). Nitric oxide synthesis inhibition promotes renal production of carbon monoxide. Hypertension.

[54] Rodriguez F., Zhang F., Dinocca S., Nasjletti A. (2003). Nitric oxide synthesis influences the renal vascular response to heme oxygenase inhibition. Am. J. Physiol. Ren. Physiol..

[55] Imai T., Morita T., Shindo T., Nagai R., Yazaki Y., Kurihara H., Suematsu M., Katayama S. (2001). Vascular smooth muscle cell-directed overexpression of heme oxygenase-1 elevates blood pressure through attenuation of nitric oxide-induced vasodilation in mice. Circ. Res..

[56] Vera T., Granger J.P., Stec D.E. (2009). Inhibition of bilirubin metabolism induces moderate hyperbilirubinemia and attenuates ANG II-dependent hypertension in mice. Am. J. Physiol. Regul. Integr. Comp. Physiol..

[57] Vera T., Stec D.E. (2010). Moderate hyperbilirubinemia improves renal hemodynamics in ANG II-dependent hypertension. Am. J. Physiol. Regul. Integr. Comp. Physiol..

[58] Stec D.E., Hosick P.A., Granger J.P. (2012). Bilirubin, renal hemodynamics, and blood pressure. Front. Pharmacol..

[59] Mazzone G.L., Rigato I., Tiribelli C. (2010). Unconjugated bilirubin modulates nitric oxide production via iNOS regulation. Biosci. Trends.

[60] Fujii M., Inoguchi T., Sasaki S., Maeda Y., Zheng J., Kobayashi K., Takayanagi R. (2010). Bilirubin and biliverdin protect rodents against diabetic nephropathy by downregulating NAD(P)H oxidase. Kidney Int..

[61] Kwak J.Y., Takeshige K., Cheung B.S., Minakami S. (1991). Bilirubin inhibits the activation of superoxide-producing NADPH oxidase in a neutrophil cell-free system. Biochim. Biophys. Acta.

[62] Stocker R., Glazer A.N., Ames B.N. (1987). Antioxidant activity of albumin-bound bilirubin. Proc. Natl. Acad. Sci. USA.

[63] Guyton A.C. (1989). Roles of the kidneys and fluid volumes in arterial pressure regulation and hypertension. Chin. J. Physiol..

[64] Guyton A.C. (1990). The surprising kidney-fluid mechanism for pressure control—Its infinite gain!. Hypertension.

[65] Botros F.T., Dobrowolski L., Navar L.G. (2012). Renal heme oxygenase-1 induction with hemin augments renal hemodynamics, renal autoregulation, and excretory function. Int. J. Hypertens..

[66] Rodriguez F., Kemp R., Balazy M., Nasjletti A. (2003). Effects of exogenous heme on renal function: Role of heme oxygenase and cyclooxygenase. Hypertension.

[67] Liu H., Mount D.B., Nasjletti A., Wang W. (1999). Carbon monoxide stimulates the apical 70-pS K+ channel of the rat thick ascending limb. J. Clin. Investig..

[68] Wang S., Publicover S., Gu Y. (2009). An oxygen-sensitive mechanism in regulation of epithelial sodium channel. Proc. Natl. Acad. Sci. USA.

[69] Tongers J., Fiedler B., Konig D., Kempf T., Klein G., Heineke J., Kraft T., Gambaryan S., Lohmann S.M., Drexler H. (2004). Heme oxygenase-1 inhibition of MAP kinases, calcineurin/NFAT signaling, and hypertrophy in cardiac myocytes. Cardiovasc. Res..

[70] Hu C.M., Chen Y.H., Chiang M.T., Chau L.Y. (2004). Heme oxygenase-1 inhibits angiotensin II-induced cardiac hypertrophy in vitro and in vivo. Circulation.

[71] Foo R.S., Siow R.C., Brown M.J., Bennett M.R. (2006). Heme oxygenase-1 gene transfer inhibits angiotensin II-mediated rat cardiac myocyte apoptosis but not hypertrophy. J. Cell. Physiol..

[72] Kobayashi A., Ishikawa K., Matsumoto H., Kimura S., Kamiyama Y., Maruyama Y. (2007). Synergetic antioxidant and vasodilatory action of carbon monoxide in angiotensin II—induced cardiac hypertrophy. Hypertension.

[73] Raju V.S., Maines M.D. (1996). Renal ischemia/reperfusion up-regulates heme oxygenase-1 (HSP32) expression and increases cGMP in rat heart. J. Pharmacol. Exp. Ther..

[74] Csonka C., Varga E., Kovacs P., Ferdinandy P., Blasig I.E., Szilvassy Z., Tosaki A. (1999). Heme oxygenase and cardiac function in ischemic/reperfused rat hearts. Free Radic. Biol. Med..

[75] Yoshida T., Maulik N., Ho Y.S., Alam J., Das D.K. (2001). H(mox-1) constitutes an adaptive response to effect antioxidant cardioprotection: A study with transgenic mice heterozygous for targeted disruption of the Heme oxygenase-1 gene. Circulation.

[76] Yet S.F., Tian R., Layne M.D., Wang Z.Y., Maemura K., Solovyeva M., Ith B., Melo L.G., Zhang L., Ingwall J.S. (2001). Cardiac-specific expression of heme oxygenase-1 protects against ischemia and reperfusion injury in transgenic mice. Circ. Res..

[77] Clark J.E., Foresti R., Sarathchandra P., Kaur H., Green C.J., Motterlini R. (2000). Heme oxygenase-1-derived bilirubin ameliorates postischemic myocardial dysfunction. Am. J. Physiol. Heart Circ. Physiol..

[78] Bakrania B., Du Toit E.F., Ashton K.J., Kiessling C.J., Wagner K.H., Headrick J.P., Bulmer A.C. (2014). Hyperbilirubinemia modulates myocardial function, aortic ejection, and ischemic stress resistance in the Gunn rat. Am. J. Physiol. Heart Circ. Physiol..

[79] Bakrania B., Du Toit E.F., Ashton K.J., Wagner K.H., Headrick J.P., Bulmer A.C. (2017). Chronically elevated bilirubin protects from cardiac reperfusion injury in the male Gunn rat. Acta Physiol..

[80] Tang Y.L., Qian K., Zhang Y.C., Shen L., Phillips M.I. (2005). A vigilant, hypoxia-regulated heme oxygenase-1 gene vector in the heart limits cardiac injury after ischemia-reperfusion in vivo. J. Cardiovasc. Pharmacol. Ther..

[81] Tang Y.L., Tang Y., Zhang Y.C., Qian K., Shen L., Phillips M.I. (2004). Protection from ischemic heart injury by a vigilant heme oxygenase-1 plasmid system. Hypertension.

[82] Clark J.E., Naughton P., Shurey S., Green C.J., Johnson T.R., Mann B.E., Foresti R., Motterlini R. (2003). Cardioprotective actions by a water-soluble carbon monoxide-releasing molecule. Circ. Res..

[83] Fujimoto H., Ohno M., Ayabe S., Kobayashi H., Ishizaka N., Kimura H., Yoshida K., Nagai R. (2004). Carbon monoxide protects against cardiac ischemia--reperfusion injury in vivo via MAPK and Akt--eNOS pathways. Arterioscler. Thromb. Vasc. Biol..

[84] Guo Y., Stein A.B., Wu W.J., Tan W., Zhu X., Li Q.H., Dawn B., Motterlini R., Bolli R. (2004). Administration of a CO-releasing molecule at the time of reperfusion reduces infarct size in vivo. Am. J. Physiol. Heart Circ. Physiol..

[85] Stein A.B., Guo Y., Tan W., Wu W.J., Zhu X., Li Q., Luo C., Dawn B., Johnson T.R., Motterlini R. (2005). Administration of a CO-releasing molecule induces late preconditioning against myocardial infarction. J. Mol. Cell. Cardiol..

[86] Yeom H.J., Koo O.J., Yang J., Cho B., Hwang J.I., Park S.J., Hurh S., Kim H., Lee E.M., Ro H. (2012). Generation and characterization of human heme oxygenase-1 transgenic pigs. PLoS ONE.

[87] Hinkel R., Lange P., Petersen B., Gottlieb E., Ng J.K., Finger S., Horstkotte J., Lee S., Thormann M., Knorr M. (2015). Heme Oxygenase-1 Gene Therapy Provides Cardioprotection Via Control of Post-Ischemic Inflammation: An Experimental Study in a Pre-Clinical Pig Model. J. Am. Coll. Cardiol..

[88] Chen M., Zhou L., Ding H., Huang S., He M., Zhang X., Cheng L., Wang D., Hu F.B., Wu T. (2012). Short (GT) (n) repeats in heme oxygenase-1 gene promoter are associated with lower risk of coronary heart disease in subjects with high levels of oxidative stress. Cell Stress Chaperones.

[89] Ono K., Goto Y., Takagi S., Baba S., Tago N., Nonogi H., Iwai N. (2004). A promoter variant of the heme oxygenase-1 gene may reduce the incidence of ischemic heart disease in Japanese. Atherosclerosis.

[90] Ullrich R., Exner M., Schillinger M., Zuckermann A., Raith M., Dunkler D., Horvat R., Grimm M., Wagner O. (2005). Microsatellite polymorphism in the heme oxygenase-1 gene promoter and cardiac allograft vasculopathy. J. Heart Lung Transplant..

[91] Yachie A., Niida Y., Wada T., Igarashi N., Kaneda H., Toma T., Ohta K., Kasahara Y., Koizumi S. (1999). Oxidative stress causes enhanced endothelial cell injury in human heme oxygenase-1 deficiency. J. Clin. Investig..

[92] Radhakrishnan N., Yadav S.P., Sachdeva A., Pruthi P.K., Sawhney S., Piplani T., Wada T., Yachie A. (2011). Human heme oxygenase-1 deficiency presenting with hemolysis, nephritis, and asplenia. J. Pediatr. Hematol. Oncol..

[93] Demirogullari B., Ekingen G., Guz G., Bukan N., Erdem O., Ozen I.O., Memis L., Sert S. (2006). A comparative study of the effects of hemin and bilirubin on bilateral renal ischemia reperfusion injury. Nephron Exp. Nephrol..

[94] Toda N., Takahashi T., Mizobuchi S., Fujii H., Nakahira K., Takahashi S., Yamashita M., Morita K., Hirakawa M., Akagi R. (2002). Tin chloride pretreatment prevents renal injury in rats with ischemic acute renal failure. Crit. Care Med..

[95] Nath K.A., Balla G., Vercellotti G.M., Balla J., Jacob H.S., Levitt M.D., Rosenberg M.E. (1992). Induction of heme oxygenase is a rapid, protective response in rhabdomyolysis in the rat. J. Clin. Investig..

[96] Vogt B.A., Alam J., Croatt A.J., Vercellotti G.M., Nath K.A. (1995). Acquired resistance to acute oxidative stress. Possible role of heme oxygenase and ferritin. Lab. Investig..

[97] Agarwal A., Balla J., Alam J., Croatt A.J., Nath K.A. (1995). Induction of heme oxygenase in toxic renal injury: A protective role in cisplatin nephrotoxicity in the rat. Kidney Int..

[98] Shiraishi F., Curtis L.M., Truong L., Poss K., Visner G.A., Madsen K., Nick H.S., Agarwal A. (2000). Heme oxygenase-1 gene ablation or expression modulates cisplatin-induced renal tubular apoptosis. Am. J. Physiol. Ren. Physiol..

[99] Bolisetty S., Traylor A., Joseph R., Zarjou A., Agarwal A. (2016). Proximal tubule-targeted heme oxygenase-1 in cisplatin-induced acute kidney injury. Am. J. Physiol. Ren. Physiol..

[100] Bolisetty S., Traylor A.M., Kim J., Joseph R., Ricart K., Landar A., Agarwal A. (2010). Heme oxygenase-1 inhibits renal tubular macroautophagy in acute kidney injury. J. Am. Soc. Nephrol..

[101] Leaf D.E., Body S.C., Muehlschlegel J.D., McMahon G.M., Lichtner P., Collard C.D., Shernan S.K., Fox A.A., Waikar S.S. (2016). Length Polymorphisms in Heme Oxygenase-1 and AKI after Cardiac Surgery. J. Am. Soc. Nephrol..

[102] Zager R.A., Johnson A.C., Becker K. (2012). Plasma and urinary heme oxygenase-1 in AKI. J. Am. Soc. Nephrol..

[103] Adin C.A., Croker B.P., Agarwal A. (2005). Protective effects of exogenous bilirubin on ischemia-reperfusion injury in the isolated, perfused rat kidney. Am. J. Physiol. Ren. Physiol..

[104] Kirkby K., Baylis C., Agarwal A., Croker B., Archer L., Adin C. (2007). Intravenous bilirubin provides incomplete protection against renal ischemia-reperfusion injury in vivo. Am. J. Physiol. Ren. Physiol..

[105] Han S.S., Na K.Y., Chae D.W., Kim Y.S., Kim S., Chin H.J. (2010). High serum bilirubin is associated with the reduced risk of diabetes mellitus and diabetic nephropathy. Tohoku J. Exp. Med..

[106] Stec D.E., John K., Trabbic C.J., Luniwal A., Hankins M.W., Baum J., Hinds T.D. (2016). Bilirubin Binding to PPARalpha Inhibits Lipid Accumulation. PLoS ONE.

[107] Goebel U., Siepe M., Schwer C.I., Schibilsky D., Foerster K., Neumann J., Wiech T., Priebe H.J., Schlensak C., Loop T. (2010). Inhaled carbon monoxide prevents acute kidney injury in pigs after cardiopulmonary bypass by inducing a heat shock response. Anesth. Analg..

[108] Neto J.S., Nakao A., Kimizuka K., Romanosky A.J., Stolz D.B., Uchiyama T., Nalesnik M.A., Otterbein L.E., Murase N. (2004). Protection of transplant-induced renal ischemia-reperfusion injury with carbon monoxide. Am. J. Physiol. Ren. Physiol..

[109] Vera T., Henegar J.R., Drummond H.A., Rimoldi J.M., Stec D.E. (2005). Protective effect of carbon monoxide-releasing compounds in ischemia-induced acute renal failure. J. Am. Soc. Nephrol..

[110] Tayem Y., Johnson T.R., Mann B.E., Green C.J., Motterlini R. (2006). Protection against cisplatin-induced nephrotoxicity by a carbon monoxide-releasing molecule. Am. J. Physiol. Ren. Physiol..

[111] Stec D.E., Bishop C., Rimoldi J.M., Poreddy S.R., Vera T., Salahudeen A.K. (2007). Carbon monoxide (CO) protects renal tubular epithelial cells against cold-rewarm apoptosis. Ren. Fail..

[112] Neto J.S., Nakao A., Toyokawa H., Nalesnik M.A., Romanosky A.J., Kimizuka K., Kaizu T., Hashimoto N., Azhipa O., Stolz D.B. (2006). Low-dose carbon monoxide inhalation prevents development of chronic allograft nephropathy. Am. J. Physiol. Ren. Physiol..

[113] Nakao A., Faleo G., Nalesnik M.A., Seda-Neto J., Kohmoto J., Murase N. (2009). Low-dose carbon monoxide inhibits progressive chronic allograft nephropathy and restores renal allograft function. Am. J. Physiol. Ren. Physiol..

[114] Bagul A., Hosgood S.A., Kaushik M., Nicholson M.L. (2008). Carbon monoxide protects against ischemia-reperfusion injury in an experimental model of controlled nonheartbeating donor kidney. Transplantation.

[115] Faleo G., Neto J.S., Kohmoto J., Tomiyama K., Shimizu H., Takahashi T., Wang Y., Sugimoto R., Choi A.M., Stolz D.B. (2008). Carbon monoxide ameliorates renal cold ischemia-reperfusion injury with an upregulation of vascular endothelial growth factor by activation of hypoxia-inducible factor. Transplantation.

[116] Ruan Y., Wang L., Zhao Y., Yao Y., Chen S., Li J., Guo H., Ming C., Chen S., Gong F. (2014). Carbon monoxide potently prevents ischemia-induced high-mobility group box 1 translocation and release and protects against lethal renal ischemia-reperfusion injury. Kidney Int..

[117] Zheng M., Zhang Q., Joe Y., Kim S.K., Uddin M.J., Rhew H., Kim T., Ryter S.W., Chung H.T. (2013). Carbon monoxide-releasing molecules reverse leptin resistance induced by endoplasmic reticulum stress. Am. J. Physiol. Endocrinol. Metab..

[118] Joe Y., Kim S., Kim H.J., Park J., Chen Y., Park H.J., Jekal S.J., Ryter S.W., Kim U.H., Chung H.T. (2018). FGF21 induced by carbon monoxide mediates metabolic homeostasis via the PERK/ATF4 pathway. FASEB J..

[119] Correa-Costa M., Gallo D., Csizmadia E., Gomperts E., Lieberum J.L., Hauser C.J., Ji X., Wang B., Camara N.O.S., Robson S.C. (2018). Carbon monoxide protects the kidney through the central circadian clock and CD39. Proc. Natl. Acad. Sci. USA.

[120] Yoshida J., Ozaki K.S., Nalesnik M.A., Ueki S., Castillo-Rama M., Faleo G., Ezzelarab M., Nakao A., Ekser B., Echeverri G.J. (2010). Ex vivo application of carbon monoxide in UW solution prevents transplant-induced renal ischemia/reperfusion injury in pigs. Am. J. Transplant..

[121] Shin D.H., Park H.M., Jung K.A., Choi H.G., Kim J.A., Kim D.D., Kim S.G., Kang K.W., Ku S.K., Kensler T.W. (2010). The NRF2-heme oxygenase-1 system modulates cyclosporin A-induced epithelial-mesenchymal transition and renal fibrosis. Free Radic. Biol. Med..

[122] Sahin K., Tuzcu M., Gencoglu H., Dogukan A., Timurkan M., Sahin N., Aslan A., Kucuk O. (2010). Epigallocatechin-3-gallate activates Nrf2/HO-1 signaling pathway in cisplatin-induced nephrotoxicity in rats. Life Sci..

[123] Tong F., Zhou X. (2017). The Nrf2/HO-1 Pathway Mediates the Antagonist Effect of L-Arginine ON Renal Ischemia/Reperfusion Injury in Rats. Kidney Blood Press Res..

[124] Wu C.C., Huang Y.S., Chen J.S., Huang C.F., Su S.L., Lu K.C., Lin Y.F., Chu P., Lin S.H., Sytwu H.K. (2015). Resveratrol ameliorates renal damage, increases expression of heme oxygenase-1, and has anti-complement, anti-oxidative, and anti-apoptotic effects in a murine model of membranous nephropathy. PLoS ONE.

[125] Yu J.B., Shi J., Zhang Y., Gong L.R., Dong S.A., Cao X.S., Wu L.L., Wu L.N. (2015). Electroacupuncture Ameliorates Acute Renal Injury in Lipopolysaccharide-Stimulated Rabbits via Induction of HO-1 through the PI3K/Akt/Nrf2 Pathways. PLoS ONE.

[126] Vlahakis J.Z., Kinobe R.T., Bowers R.J., Brien J.F., Nakatsu K., Szarek W.A. (2006). Imidazole-dioxolane compounds as isozyme-selective heme oxygenase inhibitors. J. Med. Chem..

[127] Kinobe R.T., Ji Y., Vlahakis J.Z., Motterlini R., Brien J.F., Szarek W.A., Nakatsu K. (2007). Effectiveness of novel imidazole-dioxolane heme oxygenase inhibitors in renal proximal tubule epithelial cells. J. Pharmacol. Exp. Ther..

[128] Csongradi E., Vera T., Rimoldi J.M., Gadepalli R.S., Stec D.E. (2010). In vivo inhibition of renal heme oxygenase with an imidazole-dioxolane inhibitor. Pharmacol. Res..

